# Epigallocatechin-3-Gallate (EGCG) Mitigates Endothelial and Circulating Cells Alterations Following PLLA Electrospun Mat Placement

**DOI:** 10.3390/biomedicines10061276

**Published:** 2022-05-30

**Authors:** Carmen Ciavarella, Ilenia Motta, Santino Blando, Sabrina Valente, Fulvia Farabegoli, Maria Letizia Focarete, Mauro Gargiulo, Gianandrea Pasquinelli

**Affiliations:** 1 Department of Experimental, Diagnostic and Specialty Medicine (DIMES), University of Bologna, 40138 Bologna, Italy; ilenia.motta2@unibo.it (I.M.); santinoblando@gmail.com (S.B.); sabrina.valente2@unibo.it (S.V.); mauro.gargiulo2@unibo.it (M.G.); gianandr.pasquinelli@unibo.it (G.P.); 2 FABIT—Department of Pharmacy and Biotechnology, University of Bologna, 40126 Bologna, Italy; fulvia.farabegoli@unibo.it; 3 Department of Chemistry “Giacomo Ciamician”, University of Bologna, 40126 Bologna, Italy; marialetizia.focarete@unibo.it; 4 Vascular Surgery Unit, IRCCS Azienda Ospedaliero-Universitaria di Bologna, 40138 Bologna, Italy; 5 Subcellular Nephro-Vascular Diagnostic Program, Pathology Unit, IRCCS Azienda Ospedaliero-Universitaria di Bologna, 40138 Bologna, Italy

**Keywords:** poly-L-lactic acid (PLLA), vascular graft, calcification, inflammation, epigallo-catechin-3-gallate (EGCG)

## Abstract

Background. Synthetic vascular graft calcification is a serious complication of graft placement. Here, we analysed migration and osteogenic genes of human umbilical vein endothelial cells (HUVEC) cultured with a poly-L-lactic acid (PLLA) electrospun mat. The role of epigallo-catechin-3-gallate (EGCG) in pathogenic processes involving HUVEC and peripheral blood mononuclear cells (PBMCs) was also tested. Methods. HUVEC were cultured in indirect contact with PLLA for 48 h, with or without EGCG, and processed for mRNA expression. HUVEC proliferation, migration and osteogenic differentiation were evaluated after EGCG treatment. EGCG was also administrated to human PBMCs, to analyse proliferation and migration toward HUVEC cultured with PLLA. Results. HUVEC cultured with PLLA exhibited increased expression of SLUG, VIMENTIN, MMP-9 (migration, vascular remodelling) and RUNX-2 (osteogenic transcription factor). EGCG at 25 μM significantly reduced HUVEC migration, osteogenic differentiation, without affecting cell viability, and mitigated PLLA influence on SLUG, MMP-9, VIMENTIN and RUNX-2 expression. EGCG affected PBMC proliferation and migration toward PLLA in a transwell co-culture system with HUVEC. Conclusion. Our study suggests the pro-calcific effect of PLLA, proposing EGCG as an anti-inflammatory modulatory approach. Research efforts need to deepen PLLA-vascular wall interactions for preventing vascular graft failure.

## 1. Introduction

Synthetic vascular grafts have been developed to overcome objective limitations imposed by autologous venous grafts, mainly due to the lack of available tissues in patients affected by atherosclerotic disease [1,2]. Polytetrafluoroethylene (PTFE), polyurethane (PU) and silicone are commonly used in vascular graft procedures, carrying a discrete rate of success for grafts with a diameter > 6 mm [1,3]. However, despite the enormous advance in the field of bioengineering, the rate of complications is not negligible, especially for small grafts (diameter < 6 mm), and the search for novel biocompatible materials to ameliorate graft outcome and reduce potential complication is always challenging. Research is mostly addressed to prevent graft stenosis due to intimal hyperplasia or intravascular thrombosis, whereas the in-stent graft calcification has been often overlooked [4]. In-stent graft calcification carries a high risk for thrombosis and stenosis, representing one of the most critical complications of vascular graft placement [5]. Thus, the search for biomaterials and/or therapeutic strategies to inhibit or delay the calcification of vascular graft represents an urgent clinical that needs to be addressed. 

The mechanism responsible for graft calcification is largely unknown. One of the most intriguing mechanisms responsible of arterial calcification has been attributed to vascular cell differentiation into osteogenic progenitors, as recently reviewed [6]. This process involves resident mesenchymal progenitors (mesenchymal stem cells, MSCs) [7,8], vascular smooth muscle cells (VSMCs) [9,10] and, as recently proposed, endothelial cells (ECs) [11,12]. ECs can undergo phenotype transition into mesenchymal cells able to differentiate into the osteogenic lineage by a mechanism known as endothelial-mesenchymal transition (End-MT) [13,14]. This process can be induced by various microenvironment stimuli such as inflammation [15] and oxidative stress [16,17] conditions that commonly occur in vascular graft procedures, as suggested by the study of Jiang et al., performed on rat aortas. Here, an antioxidant approach through the incorporation of 1,8- octamethylenecitrate-co-cysteine (POCC) into vascular graft was able to reduce the calcification extent and consequent degenerative alterations [14]. 

In the present study, we explored the effects of poly-L-lactic acid (PLLA), a degradable synthetic polymer used in tissue engineering [18], on human endothelial cells, Human Umbilical Vein Endothelial Cells (HUVEC). For this purpose, HUVEC were grown as a monolayer on plastic multi-well plates and a piece of 1 cm × 1 cm of PLLA fibrous mat was placed in the well, submersed in the growth medium without being in close contact with the cell layer. The rationale to this technical strategy derives from the need to mimic the PLLA/cell interactions without a physical contact, as occurs when biomaterials-based grafts are placed during vascular surgical repair. Furthermore, we tested the role of epigallo-catechin-3-gallate (EGCG), a natural flavanol derived from green tea, endowed with antioxidant and anti-inflammatory properties [19], to mitigate the calcification process. 

## 2. Materials and Methods

### 2.1. PLLA Electrospun Mat Fabrication

PLLA (Lacea H.100-E) (average molecular weight by GPC = 8.4 × 10^4^ g/mol, polydispersity index, PDI = 1.7) was supplied by Mitsui Fine Chemicals (Dusseldorf, Germany). Dichloromethane (DCM) and dimethylformamide (DMF) were purchased from Sigma-Aldrich and used without further purification. Electrospun scaffolds were produced as previously described [20]. Briefly, a PLLA solution (20% *w*/*v* in DCM/DMF 80/20) was electrospun according to the following experimental conditions: applied voltage = 20 kV, needle to collector distance = 20 cm, flow rate = 1.2 mL/h (h), temperature = 30 °C, relative humidity = 20%. Electrospun scaffolds were kept under vacuum over P_2_O_5_ at room temperature (rt) overnight to remove residual solvents. 

### 2.2. Cell Cultures and Reagents

For the present study, Human Umbilical Vein Endothelial Cells (HUVEC) and human Peripheral Blood Mononuclear Cells (PBMCs) were used. HUVEC were cultured in Dulbecco’s Modified Eagle Medium (DMEM) enriched with 10% Foetal Bovine Serum (FBS), 1% antibiotics and 0.5% non-essential aminoacids. Cells were maintained in an incubator at 37 °C, 5% CO_2_. PBMCs were isolated from peripheral blood through Ficoll-Paque density gradient separation; briefly, buffy coat pre-diluted with Phosphate Buffer Saline (PBS) was stratified on Ficoll and centrifuged at 400× *g* for 30 min (min). Then, the white cells were recovered, and seeded on a T-75 flask in RPMI with 10% FBS, 1% antibiotics and 0.5% non-essential aminoacids. After 4 h in an incubator, cells were collected and used for experiments. All growth media, supplements and buffers were purchased from Euroclone.

The inflammatory cytokines Tumour Necrosis Factor (TNF)-α and Interleukin (IL)-1β (Peprotech, Cranbury, NJ, USA) were used at 25 ng/mL in DMEM without serum to avoid cross-reactions. EGCG (Sigma Aldrich, St. Louis, MO, USA) was used at 10–25–50 μM final concentrations in DMEM without serum. 

### 2.3. HUVEC/PLLA Cultures

**Figure 1:**
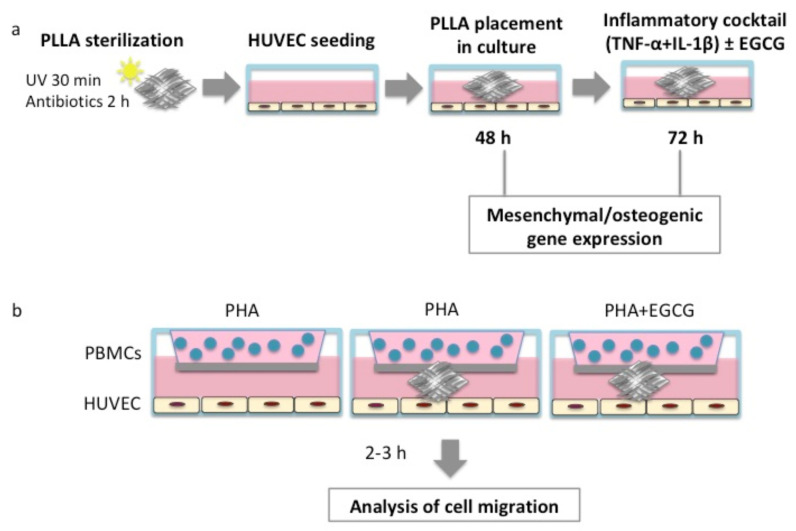
Workflow followed for PLLA/HUVEC co-culture. (**a**) PLLA slices were sterilized under UV lights for 30 min and treated with 2% antibiotics for 2 h. Then, PLLA slices were placed in the well covered by growth medium, after HUVEC attachment to the well plates. After 48 h, cells were processed for (1) gene expression, (2) EGCG testing under inflammatory conditions for 72 h, followed by gene expression analysis. (**b**) Experimental scheme of PBMCs/HUVEC co-culture in a transwell system: (1) PBMCs activated with PHA in the upper chamber and HUVEC in the lower chamber (PBMCs + PHA/HUVEC); (2) PBMCs activated with PHA in the upper chamber, HUVEC and PLLA in the lower chamber (PBMCs + PHA/HUVEC+PLLA); PBMCs activated with PHA and treated with EGCG (25 μM) in the upper chamber, HUVEC and PLLA in the lower chamber (PBMCs + PHA + EGCG/HUVEC + PLLA). After 2 h and 3 h, transwell filters were processed for the analysis of PBMC migration. EGCG: epigallo-catechin-3-gallate; HUVEC: human umbilical vein endothelial cells; PBMCs: peripheral blood mononuclear cells; PHA: phytohaemagglutinin; PLLA: poly-L-lactic acid; UV: ultraviolet.

In order to investigate whether PLLA determines perturbations on vascular wall cells, we performed an indirect culture between the polymer and HUVEC. The electrospun PLLA mat was cut with a sterile scalpel in 1 cm × 1 cm slices. For sterilization, PLLA slices were exposed to ultraviolet light (UV) radiations for 30 min and treated with 2% antibiotics for 2 h. HUVEC were seeded at a density of 8 × 10^4^ cells/well in a 24-well plate. Once cells were attached, PLLA slices were added to the culture well, submerged in culture medium, for 48 h. After co-culture, HUVEC were processed for all the experimental aims (Figure 1). 

### 2.4. Gene Expression Analysis

For the analysis of End-MT and osteogenic genes, total RNA was extracted from HUVEC through the PureZOL^TM^ RNA isolation reagent (BioRad Laboratories, Hercules, CA, USA). One μg of RNA was used for reverse-transcription in cDNA, using the iScriptTM cDNA synthesis kit (BioRad Laboratories, Hercules, CA, USA) following the manufacturer’s instructions. Real Time PCR analysis was carried on a CFX-96TM Real Time Detection System (BioRad Laboratories, Hercules, CA, USA), using the semi-quantitative Sybr Green approach (Sso AdvancedTM Universal Sybr Green Supermix; BioRad Laboratories, Hercules, CA, USA). Glyceraldehyde 3-phosphate dehydrogenase (GAPDH) was used as housekeeping gene. Specific couples of primers were designed by using the NCBI Blast Tool and the sequences are as follows: GAPDH (FWD 5′AATGGGCAGCCGTTAGGAAA 3′, REV 5′ AGGAGAAATCGGGCCAGCTA 3′), Matrix Metalloproteinase 9 (MMP-9; FWD 5′ GAACCAATCTCACCGACAG 3′, REV 5′ GCCACCCGAGTGTAACCAT 3′), Snail Family Transcriptional Repressor 2 (SLUG; FWD 5′ TTCAACGCCTCCAAAAAGCC 3′, REV 5′ GATGGGGCTGTATGCTCCTG 3′), Runt-Related Transcription Factor 2 (RUNX-2, FWD 5′ TGATGACACTGCCACCTCTGA 3′, REV 5′ GCACCTGCCTGGCTCTTCT 3′), VIMENTIN (FWD 5′ ATCGATGTGGATGTTTCCAA 3′, and REV 5′ TTGTACCATTCTTCTGCCTC 3′) (Sigma Aldrich, St Louis, MO, USA). The relative quantification was performed through the comparative DDCt method [21]. 

### 2.5. Cell Migration

The performance of HUVEC migration under treatment with EGCG was assessed through the scratch assay. Briefly, 10 × 10^4^ cells were seeded on a 24-well plate and when confluent, the monolayer was scratched with a 20 μL pipette tip. After scratch, HUVEC were treated with EGCG; then, cells were fixed with formalin for 10 min at rt. After washes with PBS, crystal violet (CV) was added to the plate for staining cells. The wound healing process was followed under the inverted light microscope, and pictures were taken at the following time: T0, when scratch was performed; T1, at 48 h; T2, at 120 h. The analysis of HUVEC migration to the wounded area was quantified by using ImageJ software. 

### 2.6. Osteogenic Differentiation

To test the effect of EGCG on the osteogenic differentiation, HUVEC were seeded at a density of 5 × 10^3^ cells/well in a 24-well plate. The osteogenic medium was composed of DMEM enriched with dexamethasone, ascorbic acid, β-glycerophosphate. EGCG was added to the osteogenic medium at 10–25–50 μM. Cells grown in DMEM 10% FBS were used as undifferentiated controls. After 14 days, HUVEC were fixed with formalin for 10 min at rt, washed with PBS and stained with Alizarin Red to mark calcium deposit, for 15 min at rt on agitation. Images were taken under phase contrast light microscope using a digital camera (Nikon). Alizarin Red was dissolved by adding 10% cetylpiridinium chloride (in Na2HPO4 10 mM, pH 7) to wells for 15 min in the dark at rt. Calcium-bound Alizarin Red was measured by reading absorbance O.D. 570 nm using a spectrophotometer. 

### 2.7. PBMC Proliferation Assay

The effect of EGCG on the inflammatory process was explored by analysing PBMC proliferation through the Bromo-deoxy-uridine (Brdu, Colorimetric Cell Proliferation Kit, Roche Applied Science, Basel, Switzerland) assay, according to the manufacturer’s instructions. Briefly, PBMCs were seeded at a density 6 × 10^5^ cells/well in a 24-well plate in RPMI 10% FBS. Phytohaemagglutinin (PHA; Gibco) was added at 1% to growth medium. PBMCs were treated with EGCG (10–25–50 μM) for 72 h and then transferred to a 96-well plate for Brdu assay.

### 2.8. Transwell Co-Culture Assay

A transwell co-culture system was established between PBMCs and HUVEC, in order to evaluate PLLA and EGCG influence on inflammatory cell migration. HUVEC were seeded at a density of 8 × 10^4^ cells/well in the lower chamber of a 24-well plate in DMEM at 10% FBS. Once cells were attached, PLLA slices were added to the culture well and submerged in culture medium for 48 h. Then, PBMCs were seeded at a density of 2.4 × 10^5^ cells/well in the upper chamber in a total RPMI volume of 400 μL, including PHA at 1%. Experimental conditions were the following: (1) PBMCs activated with PHA in the upper chamber and HUVEC in the lower chamber (PBMCs + PHA/HUVEC); (2) PBMCs activated with PHA in the upper chamber, HUVEC and PLLA in the lower chamber (PBMCs + PHA/HUVEC + PLLA); PBMCs activated with PHA and treated with EGCG (25 μM) in the upper chamber, HUVEC and PLLA in the lower chamber (PBMCs + PHA + EGCG/HUVEC + PLLA). After 2 and 3 h, the transwell insert was removed from the plate and the growth medium (including non-migrated cells) was removed by using a cotton-tipped applicator. Then, the transwell insert was fixed with 70% ethanol for 10 min, left to dry at rt and stained with 0.2% CV for 10 min at rt, following the protocol described by Justus et al. [22]. After staining, the transwell insert was rinsed in distilled water to eliminate CV in excess and left to dry. For the analysis of cell migration, the lower side of the transwell filter was observed and photographed under an inverted microscope, to detect cells undergoing the migration process. Cell count was performed on three pictures at 40x through the Cell Counter plugin of ImageJ software. 

### 2.9. Statistical Analysis

For each experiment, at least three biological and technical replicates were performed. Data analysis and graph preparation were executed through Graph Pad Prism. Statistical analysis was performed through unpaired Student’s *t*-test for comparison between two groups, one-way and two-way ordinary Anova for comparison between more than two groups. Differences were considered statistically significant at *p* value < 0.05.

## 3. Results

### 3.1. Effects of PLLA on Vascular Endothelial Cell Gene Expression

**Figure 2:**
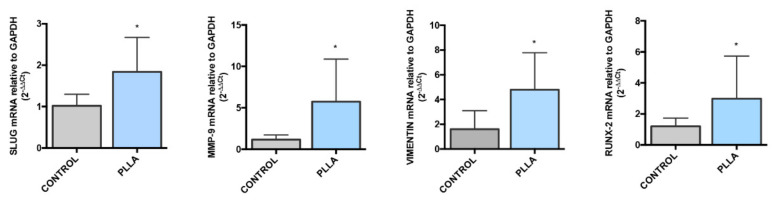
PLLA perturbs the End-MT and osteogenic phenotype in HUVEC. Gene expression analysis of SLUG, MMP-9, VIMENTIN and RUNX-2 performed by Real Time PCR in HUVEC after 48 h co-culture with PLLA. Results are reported as fold changes relative to HUVEC without PLLA (control HUVEC). All data are expressed as mean ± SD of at least 3 independent experiments, and statistical analysis was performed by unpaired Student’s *t*-test; *, *p* < 0.05. PLLA: poly-L-lactic acid; End-MT: endothelial to mesenchymal transition; HUVEC: human umbilical vein endothelial cells.

In order to test whether the PLLA induces alterations on endothelial cells, we analysed the gene expression for End-MT and osteogenic markers. We compared HUVEC grown in the presence of floating PLLA electrospun mat (PLLA-HUVEC) to HUVEC grown under traditional conditions on plastic well without PLLA mat (control HUVEC). As shown in Figure 2, we found that PLLA influences HUVEC genic signature, through the up-regulation of SLUG (1-fold increase in comparison to control HUVEC, *p* value = 0.025), MMP-9 (5-fold increase in comparison to control HUVEC, *p* value = 0.015), VIMENTIN (4 -fold increase in comparison to control HUVEC, *p* value = 0.014) and RUNX-2 (2-fold increase in comparison to control HUVEC, *p* value = 0.02). Thus, PLLA perturbs HUVEC behaviour and may trigger the osteogenic switch by End-MT differentiation. 

### 3.2. Effects of EGCG on Endothelial Proliferation and Migration

**Figure 3:**
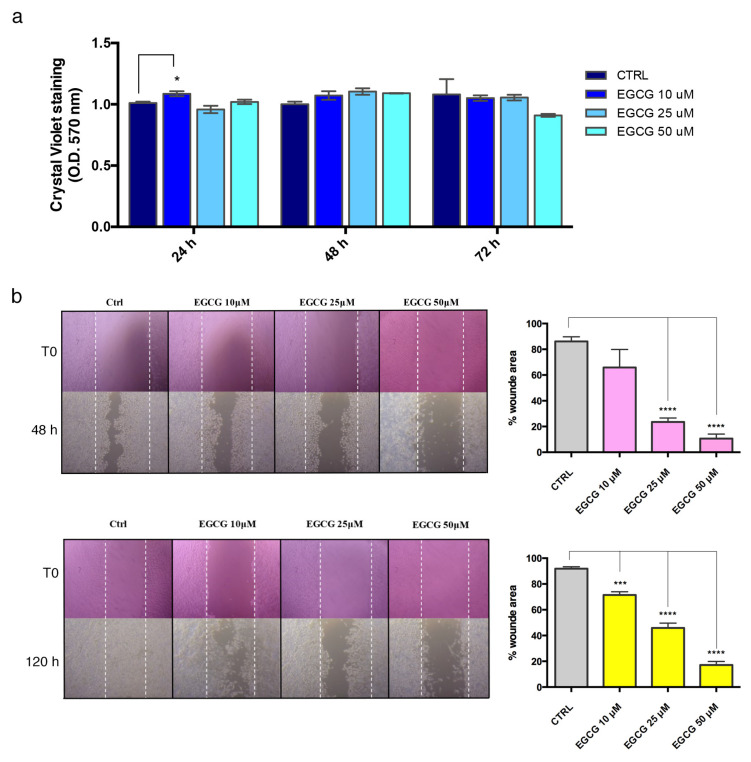
EGCG inhibits the wound healing process in HUVEC, without affecting cell proliferation. (**a**) The effect of EGCG on HUVEC proliferation was evaluated through CV stain after 24–48–72 h. (**b**) The effect of EGCG on HUVEC migration was analysed through the scratch assay. After scratch, HUVEC were treated with EGCG, and the wound healing was followed until 48 h and 120 h. For the analysis, HUVEC were fixed and stained with CV. Images are representative of three independent experiments (4× magnification). Quantification of the wounded area was measured using ImageJ software and expressed as percentage. All data are expressed as mean ± SD of at least 3 independent experiments and statistical analysis was performed by unpaired Student’s *t*-test; *, *p* < 0.05; ***, *p* < 0.001; **** *p* < 0.0001. EGCG: epigallo-catechin-3-gallate; HUVEC: human umbilical vein endothelial cells; CV: crystal violet; T0: time 0, when scratch was performed.

The effects of EGCG were preliminarily investigated on HUVEC proliferation and migration, in order to select the experimental conditions (concentration, time) for use in PLLA condition. The analysis of cell proliferation (Figure 3a), as assessed by CV staining, showed that EGCG at low concentrations (10–25 μM) does not affect HUVEC proliferation at 24 h, 48 h and 72 h. Higher EGCG amounts (50 μM) slightly decreased cell proliferation, therefore we excluded this experimental point from our study. 

Furthermore, we observed that EGCG affected the migration property of HUVEC, as supported by scratch assay analysis and quantification (Figure 3b). Interestingly, EGCG at 25 μM significantly determined a reduction in the wounded area, suggesting that this treatment can modulate HUVEC migration, a property typically observed in End-MT and occurring during stenosis of vascular grafts. 

### 3.3. Effects of EGCG on the Inflammatory Process

**Figure 4:**
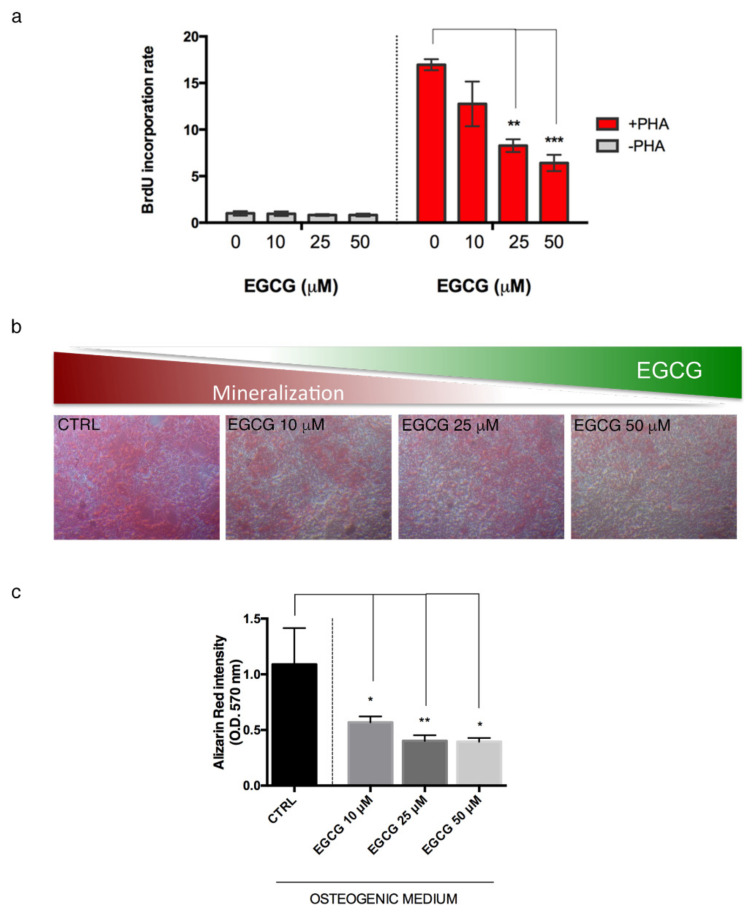
EGCG affects PBMCs proliferation and HUVEC osteogenic potential. (**a**) Analysis of Brdu incorporation rate in human PBMCs activated with PHA and treated with EGCG. Statistical analysis was performed by one-way ordinary Anova, followed by Sidak multiple comparison tests; **, *p* < 0.01; ***, *p* < 0.001. (**b**) Mineralization assay (10× magnification) and (**c**) Alizarin Red relative quantification, in HUVEC grown in osteogenic differentiation medium, and treated with EGCG. Images are representative of three independent experiments. Statistical analysis was performed by mutiple Student’s *t*-test; *, *p* < 0.05; **, *p* < 0.01. All data in this Figure are expressed as mean ± SD of at least 3 independent experiments. EGCG: epigallo-catechin-3-gallate; PBMCs: peripheral blood mononuclear cells; HUVEC: human umbilical vein endothelial cells; Brdu: bromo-deoxy-uridine.

Considering the pivotal role of the inflammatory process on vascular calcification, we investigated the effects of EGCG on PBMCs proliferation. Data obtained from the analysis of Brdu incorporation rate in PBMCs activated with PHA revealed that EGCG significantly affects PBMC proliferation in a dose-dependent manner (Figure 4a), suggesting the anti-inflammatory property of this natural compound.

### 3.4. Effects of EGCG on Endothelial Cell Calcification

Based on the clear modulating role of EGCG on a broad range of mechanisms associated with the calcification process, we investigated whether EGCG administration could influence HUVEC differentiation into the osteogenic lineage, an essential step to vascular calcification pathogenesis. Interestingly, we found a dose-dependent decrease in the mineralization property of HUVEC treated with EGCG, compared with HUVEC induced with osteogenic differentiation medium and not treated with EGCG, as clearly shown by the lower staining amount of calcium deposits through Alizarin Red (Figure 4b,c).

### 3.5. Effects of EGCG on Endothelial Cells Cultured in Presence of PLLA

**Figure 5:**
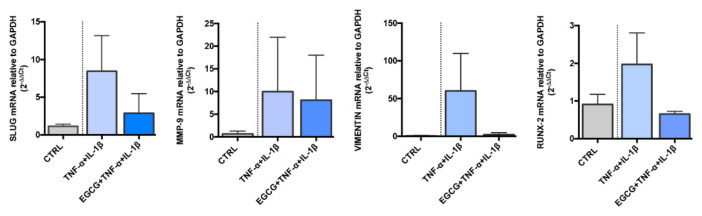
EGCG modulates the effects of inflammation in HUVEC grown with PLLA electrospun mat. Gene expression analysis of SLUG, MMP-9, VIMENTIN and RUNX-2 performed by Real Time PCR in HUVEC cultured with PLLA for 48 h, and exposed to inflammatory stimulation (TNF-α/IL-1β) ± EGCG (25 μM) addition for further 72 h. Results are reported as fold changes relative to untreated HUVEC grown in presence of PLLA. Data are expressed as mean ± SD of at least 3 independent experiments. EGCG: epigallo-catechin-3-gallate; HUVEC: human umbilical vein endothelial cells; IL-1β: interleukin 1-beta; PLLA: poly-L-lactic acid; TNF-α: tumor necrosis factor alpha.

The panel of data on effects derived from EGCG administration supported an anti-migratory, anti-inflammatory and anti-osteogenic effect on endothelial/inflammatory cells. Based on these premises, we analysed the gene expression of End-MT and osteogenic markers previously observed to increase under PLLA-HUVEC co-culture. HUVEC after 48 h of being cultured with PLLA were treated with the inflammatory cocktail TNF-α/IL-1β with or without EGCG at 25 μM for a further 72 h. We interestingly found that SLUG, VIMENTIN and RUNX-2, markedly up regulated by the inflammatory cocktail resulted affected in the presence of EGCG (Figure 5). MMP-9 did not show a significant modulation, suggesting that in this case, a higher dose of EGCG could be necessary.

### 3.6. Effects of EGCG on PBMC Migration toward HUVEC in Contact with PLLA

**Figure 6:**
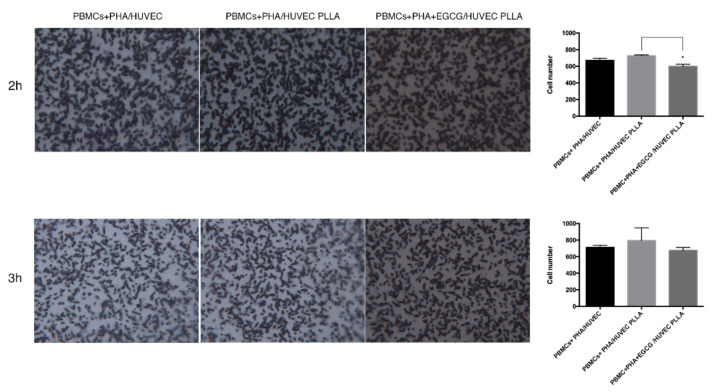
EGCG attenuates PBMC migration toward PLLA electrospun mat in a transwell co-culture system. HUVEC were cultured in presence of PLLA in the lower chamber of the transwell co-culture system for 48 h. PBMCs were seeded in the upper chamber, in RPMI medium enriched with PHA, ± EGCG (25 μM). After 2 and 3 h, the membrane was processed for CV stain and PBMC visualization under light microscope (25× magnification). Pictures and PBMC quantification are representative of three independent experiments. PBMC count was performed by using the cell counter plugin of ImageJ software. Data are expressed as mean ± SD, and statistical analysis was performed by unpaired Student’s *t*-test; *, *p* < 0.05. EGCG: epigallo-catechin-3-gallate; HUVEC: human umbilical vein endothelial cells; PBMCs: peripheral blood mononuclear cells; PHA, phytohemagglutinin; PLLA: poly-L-lactic acid.

In order to verify the influence of PLLA on the inflammatory activation contextually to stent placement, we investigated the migration of PBMCs toward the endothelial cells with/without PLLA. In a transwell-separated co-culture system, PBMCs activated with PHA were cultured in the upper chamber, whereas HUVEC were seeded on the lower chamber with PLLA, except in the control condition. After 2 and 3 h, we stained the membrane filter with CV and counted PBMCs through the cell counter plugin of ImageJ. Results are shown in Figure 6. Differences in PBMC migration were considerable after 2 h incubation. In general, PLLA increased the migration of activated PBMCs across the filter, in comparison to the control condition, where PBMCs were solely at indirect contact with HUVEC (726 cells ± 11 in PBMCs+PHA/HUVEC PLLA versus 670 cells ± 26 in PBMCs + PHA/HUVEC condition at 2 h). Interestingly, the administration of EGCG to PBMCs mitigated the rate of migrated cells reaching significant values after 2 h migration (598 cells in PBMCs+PHA+EGCG/HUVEC PLLA, *p* = 0.025, unpaired t test in comparison to PBMCs+PHA/HUVEC PLLA at 2 h; 675.5 cells in PBMCs+PHA+EGCG/HUVEC PLLA versus 794 cells ± 150 in PBMCs+PHA/HUVEC PLLA at 3 h), suggesting a modulatory role of this natural compound in the vascular cell perturbations associated with polymer-stent establishment.

## 4. Discussion

In-stent graft calcification represents a common and serious complication of vascular repair procedure. The cell players and mechanisms responsible of vascular graft failure due to the calcification process have not yet been elucidated, and few literature data are available. In the present study, we aimed to explore whether the biodegradable polymer PLLA, in the form of an electrospun mat, can modulate the End-MT process in HUVEC by altering gene expression for mesenchymal and osteogenic lineages. In our hypothesis, the inflammatory environment triggered by vascular graft procedure is a potent local stimulator of End-MT induction, following dynamics similar to those occurring during arterial calcification. Indeed, the vascular disease context is commonly characterized by inflammation, oxidative stress and hypoxia, pathological conditions that have been shown to stimulate End-MT [14]. In this regard, we recently demonstrated that End-MT induced by soluble factors, including TNF-α, TGF-β1 and TGF-β3, is associated with increased in vitro calcification [15]. In the present study, we detected an up-regulation of SLUG, VIMENTIN, MMP-9 and RUNX-2 mRNA levels in HUVEC after being cultured with PLLA for 48 h. The study of the interactions between cells and biomaterials was assessed by an indirect co-culture where a piece of PLLA electrospun mat was placed floating in the growth medium. Through this strategy, we can consider the paracrine effect exerted by PLLA on neighbouring cells. Collectively, our data suggest that PLLA modulates HUVEC behaviour, stimulating a mesenchymal and osteogenic phenotype. Accordingly, a literature study performed on the extracts of pre-degraded PLLA, demonstrated that these PLLA extracts inhibit the growth human aortic endothelial cells (HAEC) and stimulate the release of inflammatory cytokine release [23]. 

The second phase of the study was focused on EGCG, the most abundant catechin in green tea, well known for its beneficial properties. It has been shown that EGCG, in combination with the inhibitor of the retinoid X receptor (RXR), inhibits proliferation and invasive properties of different cancer cell lines [24,25]. Furthermore, EGCG exploits notable effects in vascular cells; indeed, it has been shown that EGCG reduces high-glucose vascular inflammation by inhibiting the PKC and NF-kB pathway in HUVEC [26]. Furthermore, in a study of Wu et al., a system of EGCG-loaded microparticles was developed and demonstrated an anti-inflammatory effect in human dermal fibroblasts [27]. Therefore, we aimed at investigating whether EGCG administration could ameliorate the End-MT programming in HUVEC cultured with PLLA. Firstly, we explored, with a series of functional assays, the effects of EGCG in HUVEC. Our data demonstrated the capacity of EGCG at 25 μM to significantly reduce HUVEC migration, without carrying detrimental effects on cell viability. Then, we evaluated the mineralization property of HUVEC exposed to osteogenic differentiation medium in co-treatment with EGCG. In this regard, literature data mostly support an enhancing role of EGCG in the osteogenic program, as observed in human bone marrow MSCs [28] and human periodontal ligament cells [29]. Interestingly, we found that EGCG addition to the osteogenic differentiation medium, resulted in a significant dose-dependent inhibition of HUVEC mineralization, calculated as the amount of alizarin red dye linked to calcium depositions. These results prompted us to test EGCG on HUVEC cultured with PLLA, resulting in the downregulation of SLUG, VIMENTIN, MMP-9 and RUNX-2. Thus, EGCG is effective in reducing End-MT in HUVEC, even in the presence of PLLA, as suggested by the transcriptional levels of the End-MT gene panel. In order to strengthen this hypothesis, EGCG was administrated on PBMCs activated with the mitogenic stimuli PHA, resulting in a dose-dependent decrease in cell proliferation, in accordance with the literature [30]. This result confirms the anti-inflammatory function of EGCG, and was corroborated by the analysis of cell migration in the co-culture system with HUVEC and PLLA at indirect contact for 48 h. This co-culture system was assessed to mimic the paracrine interactions among the vascular graft, endothelial cells and circulating inflammatory cells. In our hypothesis, PBMCs can be recruited both by endothelial cells with altered functions, i.e., increased permeability, induced by vascular graft, and by the vascular graft, sensed as a foreign body. In our data, we found an increase in PBMC migration in the PBMCs+PHA/HUVEC PLLA experimental condition, mitigated by EGCG treatment, as revealed by the reduced number of cells counted on the lower side of the transwell insert. 

## 5. Conclusions

Collectively, our data suggest that a commonly used polymeric material can affect vascular and inflammatory cell behaviour. In this study, PLLA stimulated End-MT in HUVEC while promoting PBMC migration. These conditions were modulated by a natural approach, through the addition of EGCG, which showed a strong potential for regulating in vitro calcification. In-stent graft calcification, such as vascular calcification, is a complex process, resulting from multiple mechanisms that are not easily regulated. The use of a natural compound, able to control and target the main cell adaptive mechanisms to inflammation, such as migration and osteogenic differentiation, can be promising. However, further functional studies are necessary to elucidate the effectiveness of this strategy and possible dual effects.

## Data Availability

The data present in this study are available within the article.

## References

[1] Tara S., Rocco K.A., Hibino N., Sugiura T., Kurobe H., Breuer C.K., Shinoka T. (2014). Vessel Bioengineering. Circ. J..

[2] Ravi S., Chaikof E.L. (2010). Biomaterials for vascular tissue engineering. Regen. Med..

[3] Park J.-C., Song M.J., Hwang Y.S., Suh H. (2001). Calcification Comparison of Polymers for Vascular Graft. Yonsei Med. J..

[4] Jiang B., Suen R., Wang J.-J., Zhang Z.J., Wertheim J.A., Ameer G.A. (2017). Vascular scaffolds with enhanced antioxidant activity inhibit graft calcification. Biomaterials.

[5] Sugiura T., Tara S., Nakayama H., Yi T., Lee Y.-U., Shoji T., Breuer C., Shinoka T. (2016). Fast-degrading bioresorbable arterial vascular graft with high cellular infiltration inhibits calcification of the graft. J. Vasc. Surg..

[6] Jiang W., Zhang Z., Li Y., Chen C., Yang H., Lin Q., Hu M., Qin X. (2021). The Cell Origin and Role of Osteoclastogenesis and Osteoblastogenesis in Vascular Calcification. Front. Cardiovasc. Med..

[7] Leszczynska A., O’Doherty A., Farrell E., Pindjakova J., O’Brien F.J., O’Brien T., Barry F., Murphy M. (2016). Differentiation of Vascular Stem Cells Contributes to Ectopic Calcification of Atherosclerotic Plaque. Stem Cells.

[8] Ciavarella C., Gallitto E., Ricci F., Buzzi M., Stella A., Pasquinelli G. (2017). The crosstalk between vascular MSCs and inflammatory mediators determines the pro-calcific remodelling of human atherosclerotic aneurysm. Stem Cell Res. Ther..

[9] Cai T., Sun D., Duan Y., Wen P., Dai C., Yang J., He W. (2016). WNT/β-catenin signaling promotes VSMCs to osteogenic transdifferentiation and calcification through directly modulating Runx2 gene expression. Exp. Cell Res..

[10] Kurozumi A., Nakano K., Yamagata K., Okada Y., Nakayamada S., Tanaka Y. (2019). IL-6 and sIL-6R induces STAT3-dependent differentiation of human VSMCs into osteoblast-like cells through JMJD2B-mediated histone demethylation of RUNX2. Bone.

[11] Yao Y., Jumabay M., Ly A., Radparvar M., Cubberly M.R., Boström K.I. (2013). A Role for the Endothelium in Vascular Calcification. Circ. Res..

[12] Sánchez-Duffhues G., De Vinuesa A.G., Van De Pol V., Geerts M.E., De Vries M.R., Janson S.G.T., van Dam H., Lindeman J.H., Goumans M.J., Dijke P.T. (2018). Inflammation induces endothelial-to-mesenchymal transition and promotes vascular calcification through downregulation of BMPR2. J. Pathol..

[13] Sanchez-Duffhues G., Orlova V., Dijke P.T. (2015). In Brief: Endothelial-to-mesenchymal transition. J. Pathol..

[14] Hao Y.-M., Yuan H.-Q., Ren Z., Qu S.-L., Liu L.-S., Wei D.H., Yin K., Fu M., Jiang Z.-S. (2018). Endothelial to mesenchymal transition in atherosclerotic vascular remodeling. Clin. Chim. Acta.

[15] Ciavarella C., Motta I., Vasuri F., Fittipaldi S., Valente S., Pollutri D., Ricci F., Gargiulo M., Pasquinelli G. (2021). Involvement of miR-30a-5p and miR-30d in Endothelial to Mesenchymal Transition and Early Osteogenic Commitment under Inflammatory Stress in HUVEC. Biomolecules.

[16] Byon C.H., Javed A., Dai Q., Kappes J.C., Clemens T.L., Darley-Usmar V.M., McDonald J.M., Chen Y. (2008). Oxidative Stress Induces Vascular Calcification through Modulation of the Osteogenic Transcription Factor Runx2 by AKT Signaling. J. Biol. Chem..

[17] Guzik T.J., Touyz R.M. (2017). Oxidative Stress, Inflammation, and Vascular Aging in Hypertension. Hypertension.

[18] Fiqrianti I.A., Widiyanti P., Manaf M.A., Savira C.Y., Cahyani N.R., Bella F.R. (2018). Poly-L-lactic Acid (PLLA)-Chitosan-Collagen Electrospun Tube for Vascular Graft Application. J. Funct. Biomater..

[19] Xie H., Sun J., Chen Y., Zong M., Li S., Wang Y. (2015). EGCG Attenuates Uric Acid-Induced Inflammatory and Oxidative Stress Responses by Medicating the NOTCH Pathway. Oxid. Med. Cell. Longev..

[20] Foroni L., Dirani G., Gualandi C., Focarete M.L., Pasquinelli G. (2010). Paraffin Embedding Allows Effective Analysis of Proliferation, Survival, and Immunophenotyping of Cells Cultured on Poly(l-Lactic Acid) Electrospun Nanofiber Scaffolds. Tissue Eng. Part C Methods.

[21] Livak K.J., Schmittgen T.D. (2001). Analysis of relative gene expression data using real-time quantitative PCR and the 2^−ΔΔCT^ Method. Methods.

[22] Justus C.R., Leffler N., Ruiz-Echevarria M., Yang L.V. (2014). In vitro Cell Migration and Invasion Assays. J. Vis. Exp..

[23] Chen D., Su Z., Weng L., Cao L., Chen C., Zeng S., Zhang S., Wu T., Hu Q., Xiao J. (2018). Effect of inflammation on endothelial cells induced by poly-L-lactic acid degradation in vitro and in vivo. J. Biomater. Sci. Polym. Ed..

[24] Farabegoli F., Govoni M., Ciavarella C., Orlandi M., Papi A. (2014). A RXR Ligand 6-OH-11-O-Hydroxyphenanthrene with Antitumour Properties Enhances (−)-Epigallocatechin-3-gallate Activity in Three Human Breast Carcinoma Cell Lines. BioMed Res. Int..

[25] Papi A., Govoni M., Ciavarella C., Spisni E., Orlandi M., Farabegoli F. (2016). Epigallocatechin-3-gallate Increases RXRγ-mediated Pro-apoptotic and Anti-invasive Effects in Gastrointestinal Cancer Cell Lines. Curr. Cancer Drug Targets.

[26] Yang J., Han Y., Chen C., Sun H., He D., Guo J., Jiang B., Zhou L., Zeng C. (2013). EGCG attenuates high glucose-induced endothelial cell inflammation by suppression of PKC and NF-κB signaling in human umbilical vein endothelial cells. Life Sci..

[27] Wu Y.R., Choi H.J., Kang Y.G., Kim J.K., Shin J.-W. (2017). In vitro study on anti-inflammatory effects of epigallocatechin-3-gallate-loaded nano- and microscale particles. Int. J. Nanomed..

[28] Lin S.-Y., Kang L., Wang C.-Z., Huang H.H., Cheng T.-L., Huang H.-T., Lee M.-J., Lin Y.-S., Ho M.-L., Wang G.-J. (2018). (−)-Epigallocatechin-3-Gallate (EGCG) Enhances Osteogenic Differentiation of Human Bone Marrow Mesenchymal Stem Cells. Molecules.

[29] Liu J., Lu Y., Liu J., Jin C., Meng Y., Pei D. (2019). Influence of epigallocatechin-3-gallate in promoting proliferation and osteogenic differentiation of human periodontal ligament cells. BMC Oral Health.

[30] Saleh F., Raghupathy R., Asfar S., Oteifa M., Al-Saleh N. (2014). Analysis of the effect of the active compound of green tea (EGCG) on the proliferation of peripheral blood mononuclear cells. BMC Complement. Altern. Med..

